# Integrative Analyses of Genes Associated With Otologic Disorders in Turner Syndrome

**DOI:** 10.3389/fgene.2022.799783

**Published:** 2022-02-22

**Authors:** Ruoyan Xue, Qi Tang, Yongli Zhang, Mengyao Xie, Chen Li, Shu Wang, Hua Yang

**Affiliations:** Department of Otolaryngology, Peking Union Medical College Hospital, Chinese Academy of Medical Sciences and Peking Union Medical College, Beijing, China

**Keywords:** turner syndrome, otologic disorders, hub genes, regulatory network, biomarker, immune infiltration, bioinformatics, enrichment analyses

## Abstract

**Background:** Loss or partial loss of one X chromosome induces Turner syndrome (TS) in females, causing major medical concerns, including otologic disorders. However, the underlying genetic pathophysiology of otologic disorders in TS is mostly unclear.

**Methods:** Ear-related genes of TS (TSEs) were identified by analyzing differentially expressed genes (DEGs) in two Gene Expression Omnibus (GEO)-derived expression profiles and ear-genes in the Comparative Toxicogenomic Database (CTD). Subsequently, Gene Ontology (GO), Kyoto Encyclopedia of Genes and Genomes (KEGG), and Disease Ontology (DO) analyses; Gene Set Enrichment Analysis (GSEA); and Gene Set Variation Analysis (GSVA) were adopted to study biological functions. Moreover, hub genes within the TSEs were identified by assessing protein-protein interaction (PPI), gene-microRNA, and gene-transcription factor (TF) networks. Drug-Gene Interaction Database (DGIdb) analysis was performed to predict molecular drugs for TS. Furthermore, three machine-learning analysis outcomes were comprehensively compared to explore optimal biomarkers of otologic disorders in TS. Finally, immune cell infiltration was analyzed.

**Results:** The TSEs included 30 significantly upregulated genes and 14 significantly downregulated genes. Enrichment analyses suggested that TSEs play crucial roles in inflammatory responses, phospholipid and glycerolipid metabolism, transcriptional processes, and epigenetic processes, such as histone acetylation, and their importance for inner ear development. Subsequently, we described three hub genes in the PPI network and confirmed their involvement in Wnt/β-catenin signaling pathway and immune cell regulation and roles in maintaining normal auditory function. We also constructed gene-microRNA and gene-TF networks. A novel biomarker (SLC25A6) of the pathogenesis of otologic disorders in TS was identified by comprehensive comparisons of three machine-learning analyses with the best predictive performance. Potential therapeutic agents in TS were predicted using the DGIdb. Immune cell infiltration analysis showed that TSEs are related to immune-infiltrating cells.

**Conclusion:** Overall, our findings have deepened the understanding of the pathophysiology of otologic disorders in TS and made contributions to present a promising biomarker and treatment targets for in-depth research.

## Introduction

Turner syndrome (TS) is a genetic disorder first proposed by Henry Turner in 1938. This syndrome occurs when an X chromosome is completely or partially absent in a female, and it has an occurrence rate of 1 in 2,500 live-born females (Sybert and McCauley, 2004). Although the prominent clinical features of TS are short stature (≤150 cm) and gonadal dysgenesis (Ranke and Saenger, 2001), the clinical characters of TS are heterogeneous, including cardiovascular, endocrine, autoimmune and neurocognition disorders (Kosteria and Kanaka-Gantenbein, 2018; Gravholt et al., 2019). It was recently reported that the otologic disorder is high incidence in the TS and is also considered as the TS dysmorphic characteristics (Dhooge et al., 2005). A serious issue in younger TS patients, recurrent otitis media can lead to conductive hearing loss, affecting nearly 88% of patients (McCarthy and Bondy, 2008; Parkin and Walker, 2009). More than one-half of adult females with TS suffer from progressive sensorineural hearing loss (SNHL), which severely influences their welfare and quality of life (Roush et al., 2000; Sybert and McCauley, 2004). However, diagnosis of this condition is often delayed. Therefore, it is important to elucidate the genetic pathogenesis of otologic disorders and explore potential biomarkers as well as prognostic indicators for accurate diagnosis and intervention.

Females with TS may have different karyotypes. Approximately one-half of TS patients have a 45, XO karyotype, 20–30% have mosaicism (45, XO/46, XX), and the remaining patients have structural abnormalities in an X chromosome (El-Mansoury et al., 2007; Gravholt et al., 2017). Because of the diversified symptoms and conditions among females with the same karyotype, many investigations have been performed to understand TS, and through these studies, the haploinsufficiency of the X-chromosome but also the epigenetic effects have been supposed with the contribution to gene expression differences (Gravholt et al., 2019). Although existing studies have reported pathophysiological hypothesis of the underlying otologic disorders in TS, including estrogen deficiency (Süsens et al., 2000; Stenberg et al., 2001), the SHOX gene hypothesis (Ross et al., 2001; Denes et al., 2015), IGF-1 decreasing (León et al., 1995; Murillo-Cuesta et al., 2011) and the KDM6A gene disturbance (Cook et al., 2015; Trolle et al., 2016), research conducted on ear and hearing is still limited. In-depth studies are needed to continuously identify possible novel candidate genes and to deepen the understanding of the pathophysiological mechanisms in TS, especially mechanisms related to ear and hearing issues.

High-throughput sequencing (HTS), or next-generation sequencing (NGS), and integrated bioinformatics analysis are effective in identifying signatures of gene expression, biological processes, and promising TS targets (Mullighan et al., 2007). A careful examination of a leukocyte methylation profile with whole transcriptome RNA sequencing (RNA-seq) data revealed that the TS genome is universally hypomethylated, with fewer hypermethylated areas than in people without TS, and this hypomethylation is accompanied by subtle differences in gene expression, particularly the expression of genes that are unaffected by X chromosome inactivation (escape genes) and those in the pseudoautosomal region (PAR) (Trolle et al., 2016). Moreover, in a previous bioinformatics analysis, the author identified potential key genes in the pathogenesis of TS through a single microarray dataset (Wang et al., 2020). However, an analysis on the underlying pathogenetic mechanisms of otologic disorders in TS is lacking.

In this study, we determined ear-related genes of TS (TSEs) by merging differentially expressed genes (DEGs) in two expression profile datasets of the Gene Expression Omnibus (GEO) and ear-related genes acquired from the Comparative Toxicogenomic Database (CTD). Subsequently, Gene Ontology (GO) analysis, Kyoto Encyclopedia of Genes and Genomes (KEGG) analysis, Disease Ontology (DO) analysis, Gene Set Enrichment Analysis (GSEA) and Gene Set Variation Analysis (GSVA) were performed to study the molecular mechanisms. Moreover, a protein-protein interaction (PPI) network was constructed, and hub genes were established using the CytoHubba plug-in. Then, we utilized the NetworkAnalyst database to investigate the target genes of microRNAs (miRNAs) and those of transcription factors (TFs), and using the Drug-Gene Interaction Database (DGIdb), we predicted possible agents. Importantly, we used machine-learning (ML) analyses to identify a novel biomarker and obtain better predictive performance. Finally, the CIBERSORT tool was used to investigate the immune cell infiltration in TS and hub genes. Our understanding of the mechanisms of otologic disorders in TS can help in identifying potential biomarkers or treatment targets.

## Materials and Methods

### Data Processing and Batch Adjustment

Raw files of three registered microarray datasets [GSE46687, GSE58435 (Massingham et al., 2014), and GSE32527 (Li et al., 2012)] containing TS tissue and normal tissue samples were obtained from the GEO database (Barrett et al., 2007) through the use of the GEOquery R package (Davis and Meltzer, 2007). The number of samples in the three datasets is shown in Table 1. *Homo sapiens* was the selected species, and the microarray expression profile was chosen as the data type. The platform for the GSE46687 dataset was the GPL570 [HG-U133_Plus_2] Affymetrix Human Genome U133 Plus 2.0 Array. The GSE58435 platform was the GPL570 [HG-U133_Plus_2] Affymetrix Human Genome U133 Plus 2.0 Array. The expression profiling of the GSE32527 dataset was based on the GPL6244 [HuGene-1_0-st] Affymetrix Human Gene 1.0 ST Array [transcript (gene) version] platform. The GSE46687 and GSE58435 gene expression profiles were merged. To conduct principal component analysis (PCA), we corrected the batch effect using the ComBat function (SVA package) (Parker et al., 2014) with the normalized read counts (Raychaudhuri et al., 2000). A total of 16,343 ear-related genes were downloaded from the CTD (http://ctd.mdibl.org) (Mattingly et al., 2006).

**TABLE 1:** The basic clinical information.

GEO	Control	Case
GSE46687	10	26
GSE32527	3	7
GSE58435	5	5

### Identification of DEGs and TSEs

To evaluate the gene expression level, the limma R package was utilized to screen DEGs in the TS and normal control samples (Ritchie et al., 2015). DEGs were defined as genes in which differences in expression had an adjusted *p*-value <0.05. Among DEGs, genes with a log fold change (logFC) >1 were considered to be significantly upregulated DEGs, while genes with logFC < −1 were considered to be significantly downregulated DEGs. Finally, we screened TSEs by obtaining the intersection of DEGs and ear-related genes.

### Construction of a PPI Network

The Search Tool for the Retrieval of Interacting Genes/Proteins (STRING) website was adopted to generate a PPI network, which was visualized in Cytoscape software (Shannon et al., 2003; Szklarczyk et al., 2019). Hub genes were identified based on Maximal Clique Centrality (MCC) (Li and Xu, 2019) through the CytoHubba plug-in (Chin et al., 2014). Biological function annotation analyses of the hub genes were performed using CluePedia (Bindea et al., 2013) and ClueGO (Bindea et al., 2009) within the Cytoscape framework. In addition, through the GOSemSim software package (Yu et al., 2010), the geometric average of the semantic similarity in cellular components (CCs) and molecular functions (MFs) was used to evaluate the functional similarity between proteins.

### Gene Function and Pathway Enrichment Analysis of the TSEs

GO function annotation analysis is a typical approach for performing large-scale gene enrichment studies, with the outcomes reported in the biological process (BP), MF and CC categories (Ashburner et al., 2000). KEGG is an information database that mainly consists of genomes, biological pathways, diseases, and drugs (Kanehisa and Goto, 2000). The DO database is a comprehensive knowledge base of genes related to diseases and relationships that are similar between diseases (Bello et al., 2018).

Herein, clusterProfiler was employed to automatically complete and visualize GO terms as well as KEGG pathways enriched with TSEs (Yu et al., 2012). The R package DOSE was used to perform a DO analysis (Yu et al., 2015). A *p*-value <0.05 was considered statistically significant.

We conducted a GSEA of GO and KEGG terms utilizing the R package clusterProfiler (Yu et al., 2012). GSVA was made via the “GSVA” R package, using fifty hallmark and C5 gene sets [KEGG, WikiPathways (WP) and REACTOME gene sets] from the Molecular Signatures Database (MSigDB) (Hänzelmann et al., 2013; Liberzon et al., 2015).

### Establishment of Target Gene-miRNA and Target Gene-TF Networks

The miRNAs and TFs that controlled the expression of genes in the context of specific illnesses were analyzed by posttranscriptional interaction with the target gene. We used NetworkAnalyst (https://www.NetworkAnalyst.ca/) (Zhou et al., 2019) for the integration of miRNA databases; TarBase (Li et al., 2014), miRTarBase (Chou et al., 2018), and the TF database ENCODE were also used. The miRNA and TF networks were visualized with Cytoscape Software (Shannon et al., 2003).

### Identification of Possible Drugs for Treating DEGs

The DGIdb version 3.0.2 was used to find latent druggable targets according to the lists of mutations and altered genes implicated in TS (Cotto et al., 2018). The DGIdb was searched to make predictions on potential molecule-related drugs that interact with DEGs, and Cytoscape software was used to visualize the interaction network between drugs and genes (Shannon et al., 2003; Cotto et al., 2018).

### Identification and Validation of Hub Genes

The data were grouped into a training set (the merged GSE46687 and GSE58435 datasets including 46 females) and a validation set (the GSE32527 dataset including 10 females). The merged dataset of 46 females was stochastically grouped into a training set (34 females) and a testing one (12 females). Radiomics feature selection was performed with 3 ML algorithms, including least absolute shrinkage and selection operator (LASSO) (Alhamzawi and Ali, 2018), random forests and Boruta (RFB) (Degenhardt et al., 2019) and extreme gradient boosting (XGBoost) (Li et al., 2019). The glmnet package (Engebretsen and Bohlin, 2019), randomForest package (Alderden et al., 2018), Boruta package and XGBoost package in R were used to calculate an important score for each feature (Sauerbrei et al., 2007; Friedman et al., 2010; Yperman et al., 2020). The input variable (independent variable) was selected based on the expression of an ear-related gene in TS, and the outcome variable (binary dependent variable, 0 or 1) was the sample disease status. After evaluating the prediction performance of the 3 ML models with the test set by the area under the receiver operating characteristic (ROC) curve (AUC) (Robin et al., 2011), the most critical biomarkers were selected on the basis of the common genes obtained through all 3 ML models, and these intersecting genes were visualized in a Venn diagram. Finally, the optimal feature gene was validated with a support vector machine (SVM) model using the radial basis function (RBF) kernel in the R package e1071 (Huang et al., 2014; Kim, 2014; Jiao et al., 2017). The GSE32527 dataset was used as the validation set to avoid over-fitting the training set.

### Immune Infiltration Analysis

CIBERSORT was employed to analyze the standardized gene expression data previously obtained, and the percentages of 22 types of infiltrating immune cells were determined (Newman et al., 2019). To obtain the immune cell matrix, we used samples in the CIBERSORT output with a *p* < 0.05. In addition, a correlation heat map was generated to show the relationships between the 22 kinds of infiltrating immune cells. To highlight the differences in immune cell infiltration, we also drew violin plots. Correlation analyses were performed with the biomarkers and infiltrating immune cells.

### Statistical Analysis

All data calculations and statistical analyses were performed using R programming. The Benjamini–Hochberg method was used for multiple-testing correction. We performed false detection rate (FDR) correction to reduce the false-positive rate in various tests. The Mann–Whitney *U* test or Student’s t test was used to make comparisons between two groups of continuous variables. ROC analysis was conducted by the pROC package. The AUC was taken as a scalar measure to evaluate the performance of prognostic risk scores. In this study, two-sided statistical tests were carried out, and a *p*-value <0.05 was considered statistically significant.

## Results

### Identification of DEGs and TSEs

**FIGURE 1:**
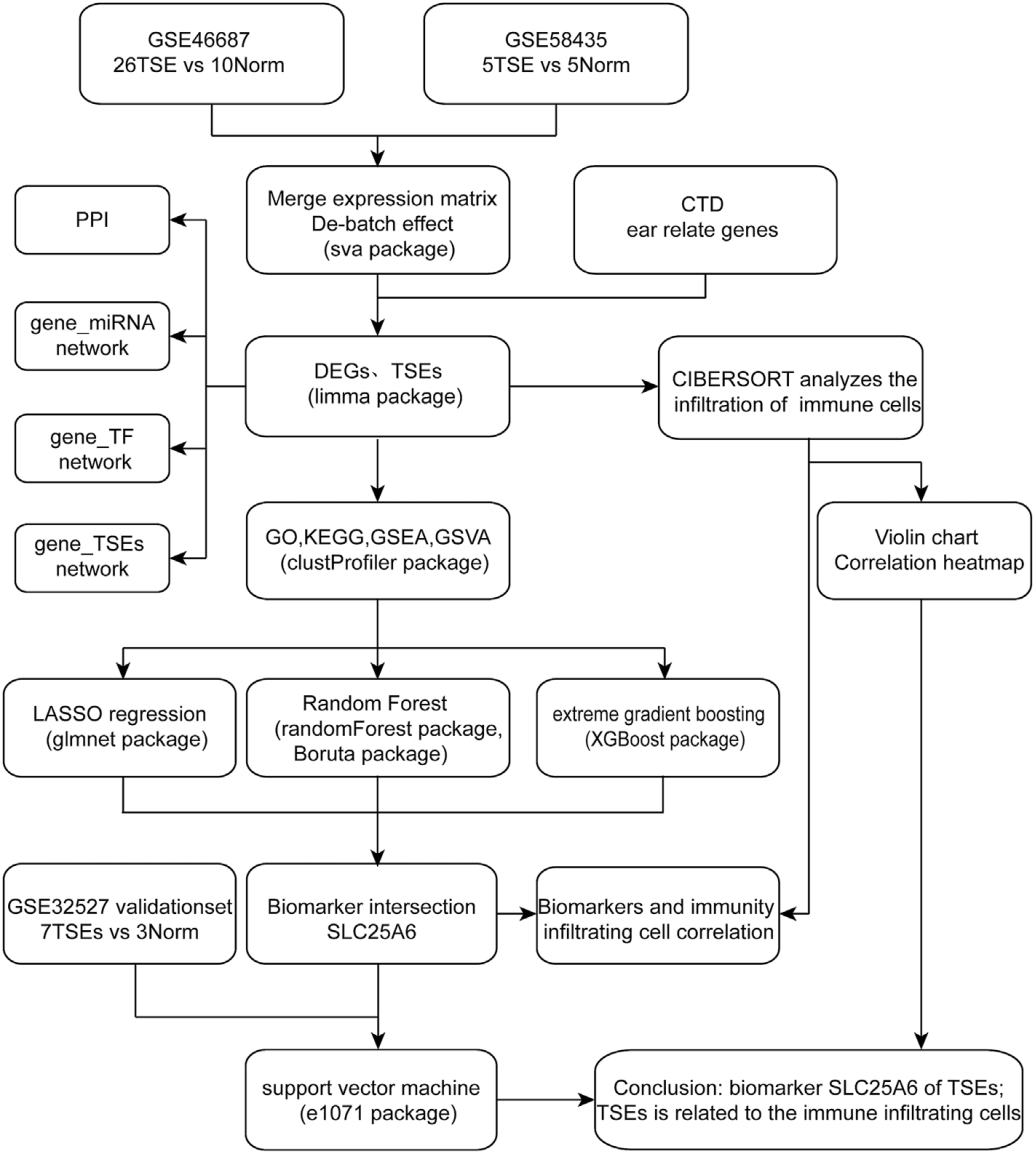
Research flow chart.

**FIGURE 2:**
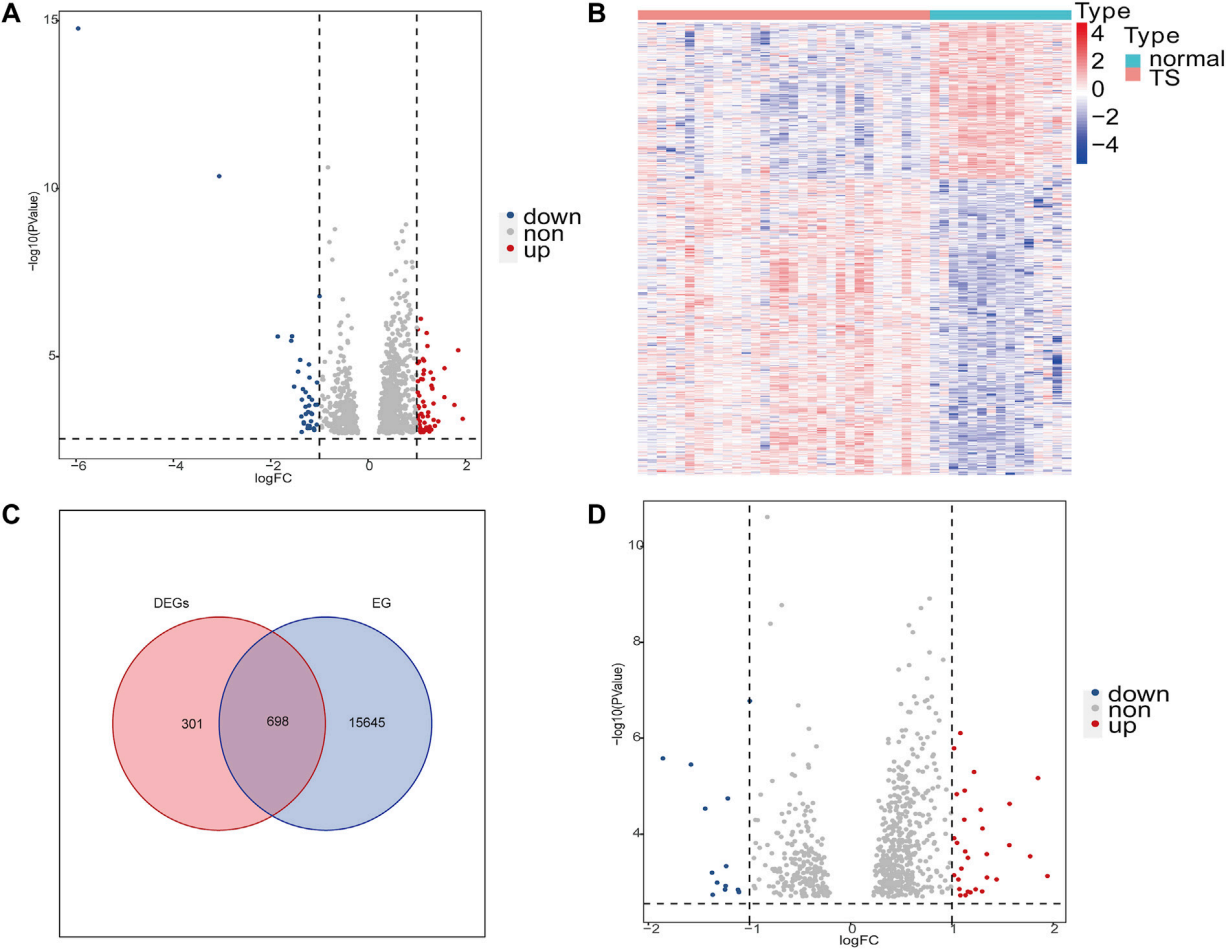
Differentially expressed genes (DEGs) of TS and ear-related genes in TS (TSEs). **(A,D)**: Volcano plot of DEGs and TSEs. The abscissa is the log2FoldChange, and the ordinate is −log10 (adjusted *p*-value). Red indicates upregulated genes, blue indicates downregulated genes, and genes with no significant expression differences are shown in gray. **(B)**: Heat map of the DEGs in TS. High expression is highlighted in red; low expression is highlighted in blue. **(C)**: Venn diagrams displaying the intersections of DEGs and ear-related genes (EGs). DEGs are red circles. Blue circles represent EGs.

The study flow chart was shown in Figure 1. After removing batch differences (Supplementary Figure S1), the two datasets were merged, and close clustering of the samples after normalization was performed through a two-dimensional PCA plot (Supplementary Figure S2), and the results showed that the sources of the samples were reliable. After data preprocessing, 999 DEGs were obtained from the matrix of gene expression, including 57 significantly upregulated DEGs and 39 significantly downregulated DEGs, as presented in the volcano plot (Figure 2A) and the heat map (Figure 2B). After intersecting the DEGs and ear-related genes, 698 TSEs were identified, including 30 significantly upregulated TSEs and 14 significantly downregulated TSEs (Figures 2C,D).

### Establishment of the PPI Network and Selection of Potential Crucial Genes

As illustrated in Supplementary Figure S3A, the PPI network of TSEs was based on the STRING database. We employed the MCC method to analyze hub genes with the Cytoscape CytoHubba plug-in. Hub genes were described as the 20 genes with the highest scores (Supplementary Figure S3B). To verify a hub gene’s function, ClueGO and CluePedia within the Cytoscape framework were utilized to perform biological function annotation analyses (Supplementary Figure S3C). In addition, according to their average functional similarity, the 10 most similar genes were selected and ranked among the 20 hub genes (Supplementary Figure S3D). RNF220, TRIM21, and STUB1 were the most highly expressed hub genes.

### Functional Enrichment Analysis

**FIGURE 3:**
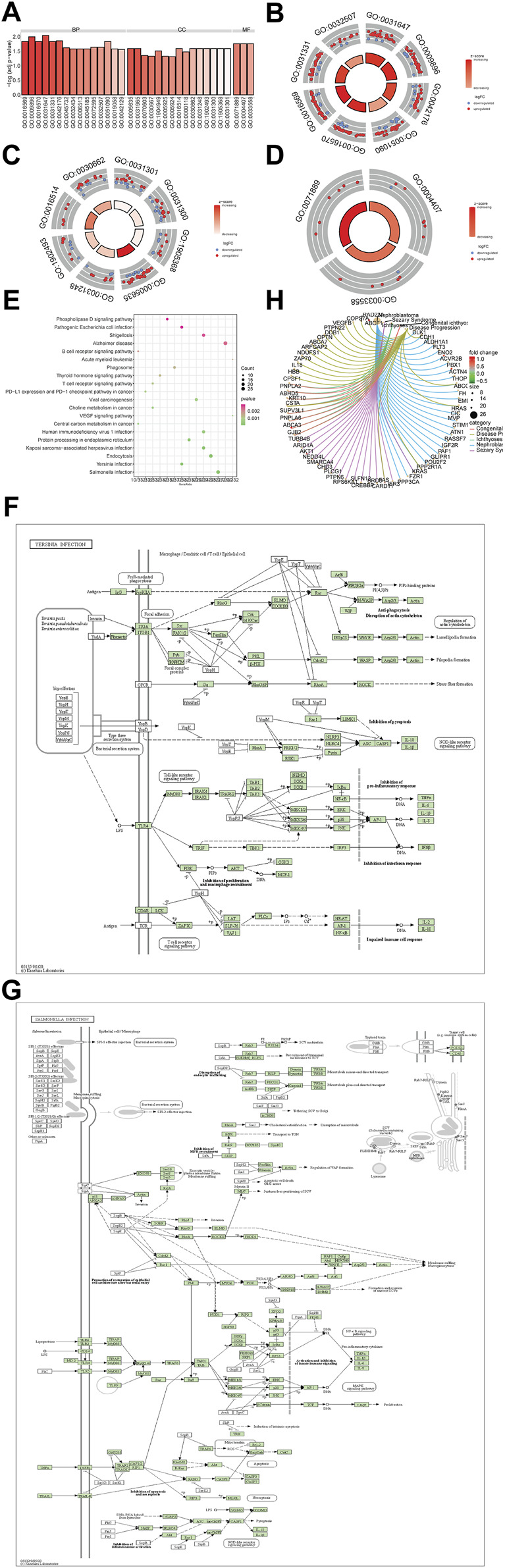
Functional enrichment analysis of the TSEs. **(A–D)**: Enrichment analysis of TSEs in the GO BP, MF, and CC categories. **(A)**: The abscissa is the z-score, and the ordinate is −log (adjusted *p*-value). **(B–D)**: The color of the node indicates the expression value: red indicates upregulated expression, and green denotes downregulated expression. The middle quadrilateral represents the effect of enriched gene expression in GO terms. Light colors indicate activation; dark colors indicate inhibition. **(E)**: KEGG pathway enrichment analysis. The abscissa is the gene ratio, and the ordinate is the pathway name. The node size represents the number of genes in the enriched pathway. The node color represents −log10 (*p*-value). **(F–G)**: The most significantly enriched KEGG pathways are displayed. **(H)**: The 5 most enriched TSE in the DO analysis are presented.

To deeply explore the biological functions of the TSEs, GO analysis was performed with Database for Annotation, Visualization, and Integrated Discovery (DAVID) online tools (Figure 3A). GO enrichment analysis results were shown in Supplementary Table S1. The BP analysis illustrated that the TSEs are mainly enriched in regulating protein stability, positively regulating catabolic processes, regulating protein catabolic processes, regulating DNA-binding transcription factor activity, modifying histones and covalent chromatin, positively regulating cellular catabolic processes, and maintaining protein location in cells (Figure 3B). The CC analysis showed that TSEs are enriched in monolithic components of the organelle membrane, complex components of the organelle membrane, the peptidase complex, the nuclear envelope, the protein acetyltransferase complex, the acetyltransferase complex, the SWI/SNF complex, and coated vesicle membranes (Figure 3C). Changes in the MF category of TSEs were significantly enriched in histone deacetylase activity, protein deacetylase activity, and 14-3-3 protein binding (Figure 3D).

To explore the potential mechanism of the TSEs, KEGG pathway analysis was performed, and the results are drawn in Figure 3E and Supplementary Table S1. It is shown that TSEs were chiefly involved in *Salmonella* infection (Figure 3F) and *Yersinia* infection (Figure 3G).

To explore the role of TSEs in diseases, a DO enrichment analysis was performed, and the results are drawn in Figure 3H and Supplementary Table S1. TSEs were significantly enriched in nephroblastoma, sezary syndrome, congenital ichthyosis, papillary neoplasm and other diseases.

### GSEA and GSVA of the TSEs

**FIGURE 4:**
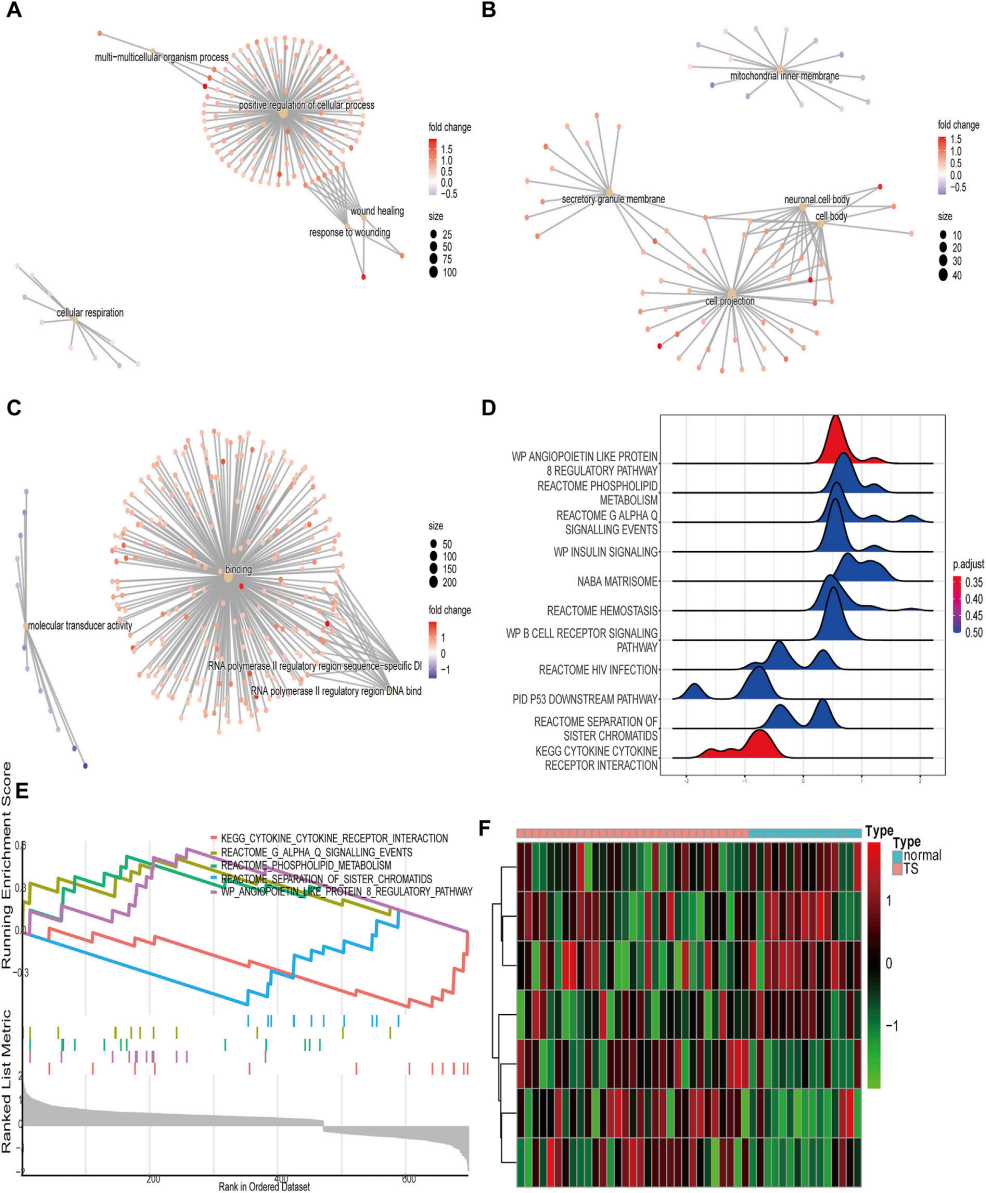
GSEA and GSVA of the TSEs. **(A–C)**: The top 5 enriched GO terms of BP, MF, and CC categories are determined by GSEA. **(D)**: Wave plot of KEGG enrichment obtained by GSEA. **(E)**: Display of the five most enriched pathways in the GSEA of the KEGG database. **(F)**: Heat map showing the enrichment scores of the hallmark ear-related TS gene sets.

To better understand the biological pathway, the functions of TSEs were predicted by performing a GSEA of the GO and KEGG results. The GSEA enrichment analysis results were shown in Supplementary Table S2. The GSEA of the GO BP category showed that TSEs were significantly enriched in the response to wounding, the glycerolipid metabolic process, wound healing, positive cellular process regulation, multicellular organism processes, and cellular respiration (Figure 4A). The GSEA of the CC category showed that TSEs were enriched in the cell body, the neuronal cell body, the mitochondrial inner membrane, cell projections, monolithic components of the membrane, and complex components of membrane (Figure 4B). The TSEs were remarkably enriched in binding, molecular transducer activity, signaling receptor activity, RNA polymerase II regulatory area sequence-specific DNA binding, RNA polymerase II regulatory area DNA binding, and transmembrane signaling receptor activity in the MF category (Figure 4C). The GSEA of KEGG pathways suggested that the most significantly enriched gene sets were involved in the angiopoietin-like protein 8 regulatory pathway (wave plot), cytokine–cytokine receptor interactions, reactome phospholipid metabolism, reactome G alpha Q signaling events, insulin signaling (wave plot), reactome separation of sister chromatids and other pathways (Figures 4D,E).

For a more detailed analysis, we performed a GSVA on the TSEs. The results revealed that seven hallmarks were differentially enriched between normal tissues and TS tissues in the following gene sets: oxidative phosphorylation, Wnt/β-catenin signaling, protein secretion, apical junction, unfolded protein response, mitotic spindle, and upregulated KRAS signaling (Figure 4F).

### Establishment of miRNA-Gene, TF–Gene, and TF-miRNA Target Networks

The 3 TSEs that are regulated by the most miRNAs were identified to be ANKRD33B, which is regulated by 86 miRNAs; PIK3R3, which is regulated by 83 miRNAs; and SGMS2, which is regulated by 76 miRNAs. The miRNAs controlling the greatest number of TSEs are hsa-mir-124-3p (Supplementary Figure S4A). The 5 genes targeted by the most TFs are AGER, which is regulated by 126 TFs; MYLPF, which is regulated by 108 TFs’ TM7SF2, which is regulated by 107 TFs; KLF16, which is regulated by 92 TFs; and HYAL3, which is regulated by 87 TFs (Supplementary Figure S4B).

The DGIdb was applied to make predictions on possible medicines or molecular compounds reacting with DEGs and construct the interaction between drugs and genes. As illustrated in the drug-gene interaction network (Supplementary Figure S4C), 29 drugs or molecular compounds, such as everolimus, lenalidomide, and ponatinib, interacted with KRAS. Furthermore, 14 drugs or molecular compounds, including imatinib, modulated FLT3, and 11 drugs or molecular compounds, including irinotecan and carboplatin, modulated AKT1.

### Screening and Verification of Biomarkers

**FIGURE 5:**
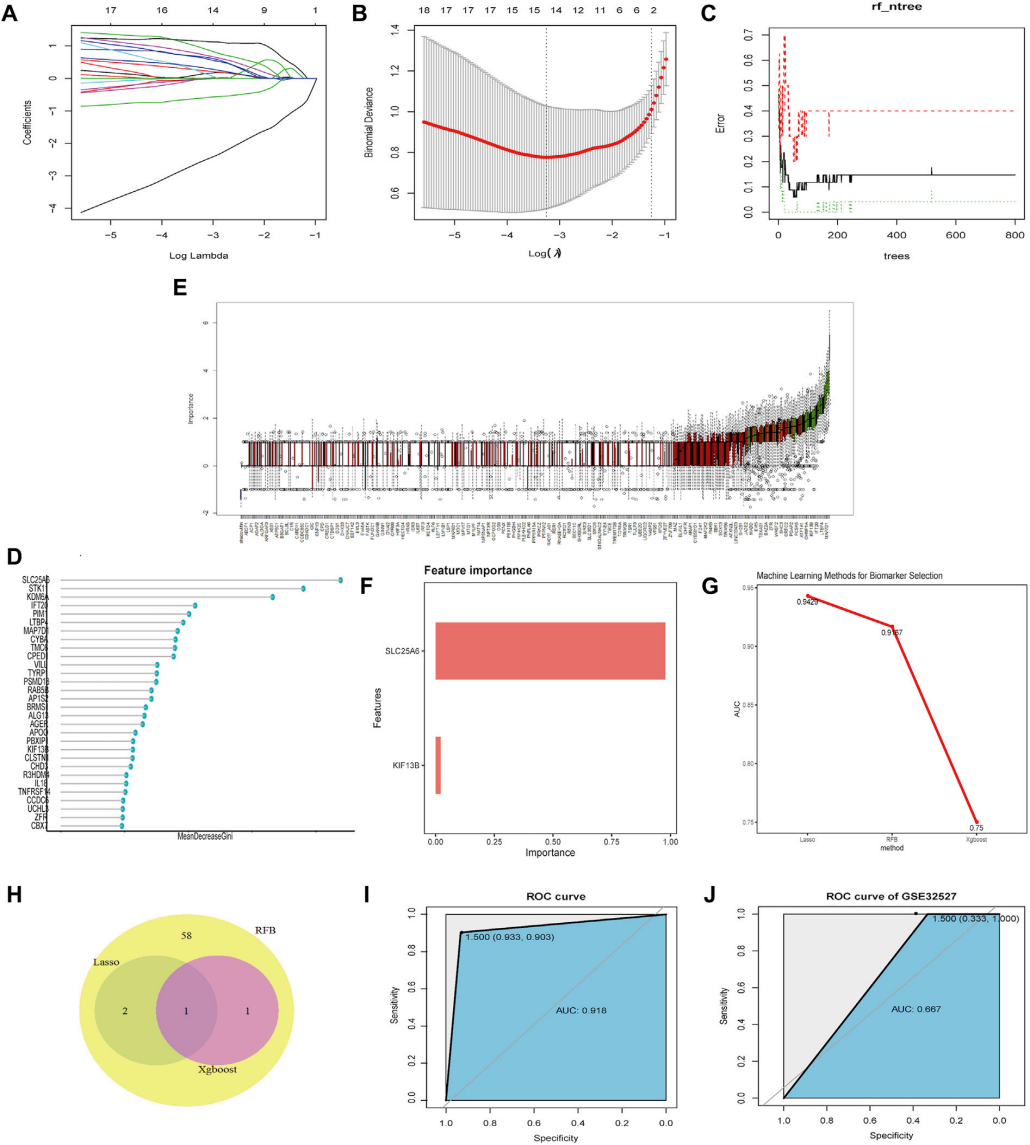
Screening and validation of biomarkers. **(A–B)**: LASSO was employed to screen biomarkers. The different colors correspond to distinct genes. **(C–E)**: The RFB algorithm was used to screen potential biomarkers. **(E)**: ROC of the hub genes useful for the diagnosis of otologic disorders in TS. **(F)**: The XGBoost algorithm was used to screen potential biomarkers. **(G)**: The feature selection performance of the three machine-learning algorithms (LASSO, RFB, and XGBoost) was evaluated with training and test sets. A ROC analysis was performed to calculate the AUC. **(H)**: Venn diagrams showing the intersection of biomarkers obtained from three machine-learning algorithms. **(I)**: ROC curve of SLC25A6 in the merged dataset. **(J)**: ROC curve of SLC25A6 in the validation dataset (GSE32527).

In the training set, 3, 62 and 2 genes were identified on the basis of LASSO, RFB and XGBoost analysis, respectively (Figures 5A–F). ROC curves showed that these 3 ML algorithms showed excellent performances on feature selection with the testing set; the AUC >0.7 (Figure 5G). Venn diagrams indicated that the shared gene in the three groups was SLC25A6 (Figure 5H). To further test the diagnostic efficiency of SLC25A6 in TS, we used an SVM with an RBF kernel (kernel = radial, cost = 100, gamma = 1) and SLC25A6 as the feature and merged the datasets to build a classification model to train the ML models. The performance evaluations were conducted using the pROC package in R. In the training set, the AUC of the SVM model was 91.8%, indicating that the model accurately distinguished the TS samples from the normal samples (Figure 5I). To prevent overfitting of the prediction model on the training set, we used the GSE32527 dataset as the validation set. The AUC of the SVM model in the GSE32527 validation set was 66.7% (Figure 5J). These results showed that SLC25A6 can be used as an effective and accurate biomarker for otologic disorders in TS.

### Infiltration of Immune Cells

**FIGURE 6:**
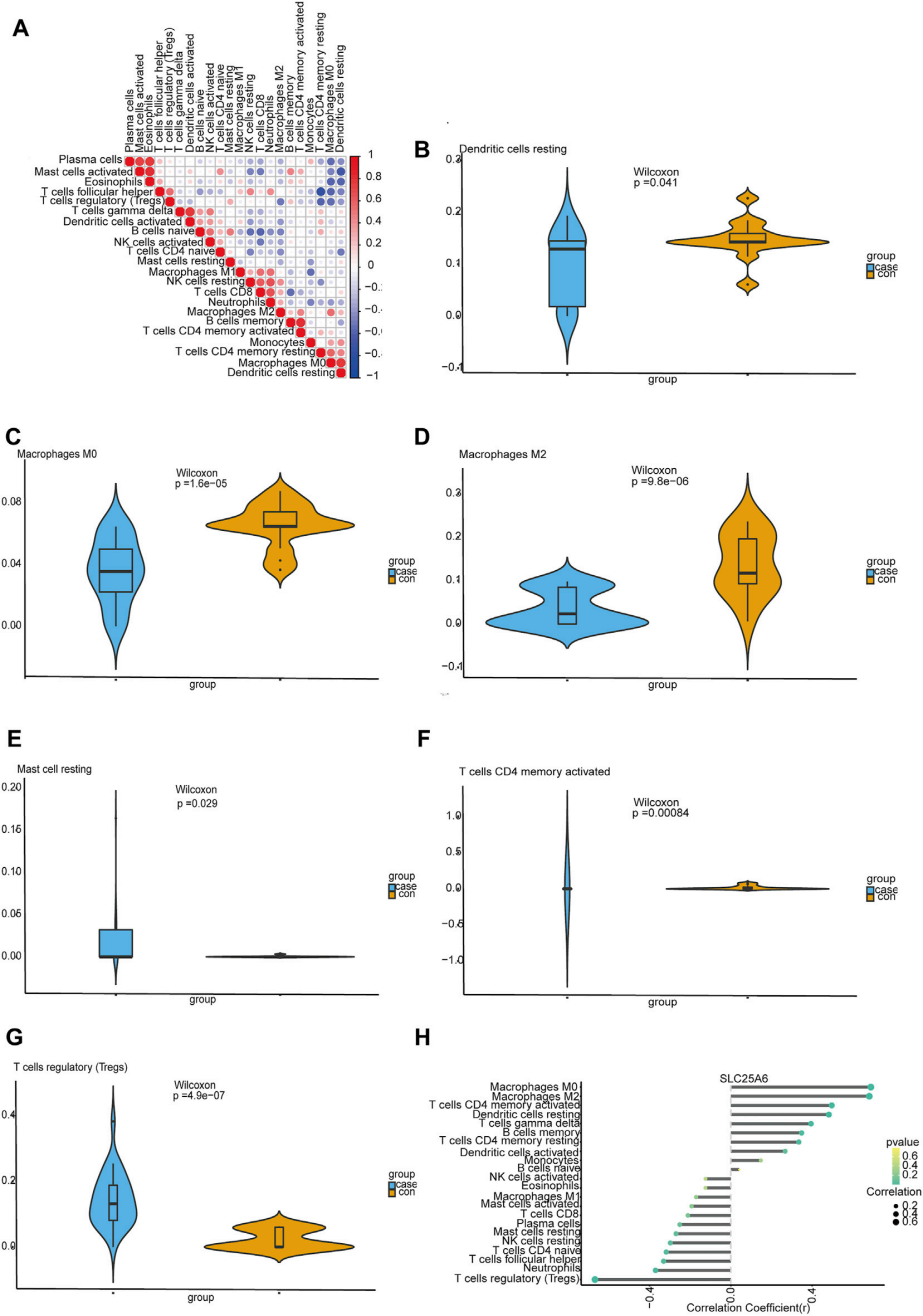
Evaluating and visualizing immune cell infiltration. **(A)**: The correlation heat map of 22 immune cells. The degrees of correlation are reflected by the size of the colored square; a positive correlation with the signature is represented by red squares, and a negative correlation is represented by blue squares; the darker the color, the stronger the correlation. **(B–G)**: Violin plots showing immune cells that are significantly different between normal and TS samples; the blue violin represents the TS samples, and the orange violin represents the normal samples. **(H)**: Correlation between SLC25A6 and the extent of immune cell infiltration. The dot sizes represents the correlation strength between genes and immune cells; the larger the dot is, the stronger the correlation. The *p*-value is denoted by dot color, where the more intense the green is, the smaller the *p*-value, and the more intense the yellow is, the larger the *p*-value. A *p*-value of 0.05 was considered to be statistically significant.

Using CIBERSORT, we investigated latent correlations between 22 kinds of immune cells. The heat map showed that the rates of different infiltrating immune cells have a weak to moderately correlated (Figure 6A). We also assessed the immune cell infiltration difference between TS and healthy tissues. The violin plot showing these data indicated significant variation in the infiltration of resting dendritic cells, M0 macrophages, M2 macrophages, resting mast cells, activated memory CD4 T cells and regulatory T cells (Tregs) (Figures 6B–G). Furthermore, the correlation analyses suggested that SLC25A6 was positively linked with B cell memory cells (*r* = 0.348, *p* = 0.018), resting CD4 memory T cells (*r* = 0.334, *p* = 0.023), activated CD4 memory T cells (*r* = 0.496, *p* = 0.0004), gamma delta T cells (*r* = 0.394, *p* = 0.006), M0 macrophages (*r* = 0.689, *p* = 1.21e-07), M2 macrophages (*r* = 0.681, *p* = 1.9e-07), and resting dendritic cells (*r* = 0.482, *p* = 0.0007). SLC25A6 was negatively linked with naïve CD4 T cells (*r* = −0.318, *p* = 0.031), follicular helper T cells (*r* = −0.331, *p* = 0.025), Tregs (*r* = −0.668, *p* = 3.86e-07), resting NK cells (*r* = −0.298, *p* = 0.044), and neutrophils (*r* = −0.370, *p* = 0.011) (Figure 6H).

## Discussion

TS is a typical chromosomal situation caused by the loss of part or all of an X chromosome in females (Lowenstein et al., 2004). Recently, many studies have proven that the incidence of secretory otitis media, cholesteatoma, and hearing loss in TS patients is remarkably higher than that of healthy people and has a younger age of onset (Roush et al., 2000; Bonnard et al., 2017). Although ear and hearing problems do not endanger the lives of TS patients, they have a certain impact on learning, psychology, and quality of life (McCarthy and Bondy, 2008; Kosteria and Kanaka-Gantenbein, 2018). Therefore, early diagnosis of TS and timely interventions are particularly important. So far, early diagnosis and intervention are hampered by the lack of valid biomarkers and poor understanding of the pathological and molecular mechanisms (Lee and Conway, 2014). With advances showing changes in epigenetic regulation, RNA expression, and PPI, the way of thinking in the genomics era has shifted from highlighting individual genes on the lost X chromosome to concentrating on changes in the genome, transcriptome, and epigenome to explain TS phenotypic changes (Gravholt et al., 2019). Effective microarray and bioinformatics analyses can help illuminate the molecular mechanisms and pathogenesis of diseases (Mullighan et al., 2007). Hence, herein, mixed bioinformatics methods were used to analyze how hub genes change in TS otologic disorders on the basis of data in two GEO datasets (GSE46687 and GSE58435) and the CTD. In total 698 TSEs were identified, containing 30 significantly upregulated TSEs and 14 significantly downregulated TSE. The enrichment analyses suggested that TSEs play a crucial role in inflammatory responses, phospholipid and glycerolipid metabolism, transcriptional processes, and epigenetic processes, such as histone acetylation, which are essential for the growth of the inner ear. Moreover, the three hub genes were determined with the highest PPI scores. Most of these genes were confirmed to be crucial regulatory genes in the Wnt/β-catenin signaling pathway and in immune cell regulation, and they play roles in ubiquitination. Then, three target TSEs were identified in the miRNA-gene network, one target gene was identified on the basis of a related miRNA in the TF-miRNA-target network and five target TSEs were identified in the TF-gene network, and they were analyzed. In addition, we identified a novel biomarker (SLC25A6) for the pathogenesis of otologic disorders in TS. Moreover, the DGIdb was used to predict possible therapeutic drugs in TS. The immune cell infiltration analysis showed that TSEs were related to infiltrating immune cells.

To analyze TS ear-related gene functions, GO, KEGG, and DO enrichment analyses were performed. In the MF category, histone deacetylase activity, protein deacetylase activity and 14-3-3 protein binding were found to have significant associations with the onset and progression of otologic disorders in TS. 14-3-3 proteins are crucial to cell signaling events. These events control cell cycle progression, transcriptional alterations responding to environmental signs, and organized cell death. In particular, 14-3-3 proteins directly modulate the activity of protein kinases and phospholipases and are known as “bridge proteins” (Thomas et al., 2005). Notably, a previous study demonstrated the expression of 14-3-3 genes in the inner ear and implicated their importance of their encoded proteins in ear development (Kandpal et al., 1997). Moreover, in the BP and CC categories, a group of GO terms were related to metabolism, regulation of proteins, epigenetic changes, transcription processes, the structure of the membrane and the SWI/SNF complex. Among BPs, epigenetic changes such as covalent chromatin modification, and especially histone modification such as acetylation, are particularly prominent functions of TSEs. Recently, the significant roles of epigenetic modifications in hearing loss, protection, and regeneration have attracted increasing attention (Layman and Zuo, 2014). In the inner ear, a histone deacetylase (HDAC) inhibitor can protect hair cells from aminoglycosides *in vitro* and cisplatin *in vivo* (Drottar et al., 2006; Chen et al., 2009). In addition, the SWI/SNF complex binds not only to promoters but also to other regulatory regions, such as enhancers and DNA replication initiation regions (Mittal and Roberts, 2020). In addition, the SWI/SNF complex can bind/coprecipitate many proteins and influence the cell cycle, cytoskeleton, chromosome organization and development of cochlear hair cells (Ahmed et al., 2012; Jin et al., 2019). Moreover, the KEGG enrichment analysis revealed that *Salmonella* infection and *Yersinia* infection were the most notably enriched pathways. *Yersinia* infection triggers the regulation of the actin cytoskeleton and NOD-like receptor signaling pathway by inhibiting pyroptosis and the proinflammatory response. *Salmonella* infection activates the NK-κB and MAPK signaling pathways. These findings may explain the reason that these TSE-associated are related to immune and inflammatory responses. Furthermore, in the DO enrichment analysis, TSE were significantly associated with nephroblastoma, sezary syndrome, congenital ichthyosis, and papillary neoplasm. This gene enrichment result may suggest that we need to reassess the risk of these diseases for people with TS.

The GSEA suggested a new perspective on the biological functions of TSE. The GSEA of GO annotation suggested that most genes in the BP category are enriched in the glycerolipid metabolic process, and cellular respiration is a key function of mitochondria and inflammation-related processes. In the CC category, the GO enrichment terms included both mitochondrial inner membrane and neuronal cell body. In the MF category, the key GO terms involved transcriptional processes, such as RNA polymerase II regulatory area sequence-specific DNA binding. In addition, the GSEA of KEGG pathways provided further evidence that TSE are mainly enriched in the angiopoietin-like protein 8 regulatory pathway, insulin signaling pathway, phospholipid metabolism, G alpha Q signaling events and separation of sister chromatids. A previous study showed the roles of the angiopoietin-like protein 8 regulatory pathway and insulin signaling pathway in enhancing glucose tolerance and insulin sensitivity under insulin resistance conditions (Siddiqa et al., 2017). Phospholipid metabolism plays a key part in physiological and pathological mechanisms in the cochlea (Peulen et al., 2019; Cunningham et al., 2020). Investigations have shown that the G alpha Q signaling event is important in the immune system and has a regulatory effect on many immune cells (McMullan et al., 2012).

The PPI network showed that RNF220, TRIM21, and STUB1 were the three most highly expressed regulatory gene candidates of otologic disorders in TS. The RNF220 gene encodes a RING domain E3 ubiquitin ligase which is a regulator of β-catenin and promotes canonical Wnt signaling, has been reported to meditate tumorigenesis in colon cancer (Ma et al., 2014). Moreover, the Wnt signaling activation causes inner ear organoids including vestibular-like hair cells to self-organize and mature (Koehler et al., 2017). The member of the tripartite motif (TRIM) family is encoded by the TRIM21 gene. The TRIM21 protein is widely expressed in various cell types, particularly in certain immune cells, like T cells, macrophages, and dendritic cells (Fletcher et al., 2015). The STUB1 gene encodes the STUB1/CHIP protein, which participates in the degradation of KCNQ4 channels and exerts a great influence on the maintenance of cochlear ion homeostasis and regulation of hair cell membrane potential. Besides, a recent study has found that a novel mechanism of Wnt/β-catenin mediated transcriptional activation of CHIP leads to enhanced proliferation of colorectal carcinoma cells (Kal et al., 2022). So these genes are essential to maintain normal auditory function and two of them are involved in Wnt/β-catenin signaling (Gao et al., 2013). Although all hub genes are located on autosomes, the loss of the X chromosome may affect autosomal gene expression, leading to differences in gene expression in the disease (Trolle et al., 2016). Overall, these three hub genes are of great importance in the inner ear and immune response, offering some new potential therapeutic targets for the disease.

The top three target TSE identified in the miRNA-gene network analysis were ANKRD33B, PIK3R3, and SGMS2. The SGMS2 protein is in the cell membrane and regulates the levels of ceramide (Cer) and sphingomyelin (SM), both of which participate in signal transduction in cells (Zheng et al., 2019). SM synthase (SMS) family can be classified into two members: SMS1 and SMS2. A study established that SMS1 is necessary for the usual function of the inner ear (Matsumoto et al., 2019). The 5 most prevalent target TSE in the TF-gene network were AGER, MYLPF, TM7SF2, KLF16, and HYAL3. AGER contributes to cholesteatoma pathogenesis by inducing cholesteatoma tissue proliferation and migration and releasing proinflammatory cytokines (Szczepanski et al., 2015).

The database of DGIdb was used to find therapeutic drugs so that the promising useful therapy for TS could be predicted. In the current study, we found that the immune system is involved in the pathogenetic mechanisms of TS and that lenalidomide activates and precisely regulates the immune system. The effects of drugs or molecular compounds in TS and the potential therapeutic targets related to comorbidities still need to be studied in depth.

Importantly, we identified a novel biomarker for the pathogenesis of TS using ML analyses. Similar to SHOX (Sybert and McCauley, 2004), the SLC25A6 gene is located in pseudoautosomal regions (PAR1) in the short subtelomeric area of the X chromosome (p-arm) and the Y chromosome (q-arm) (Schiebel et al., 1993). PARs are important for maintaining the function and structure of sex chromosomes and for guaranteeing proper pairing and isolation of the sex chromosome during meiosis (Otto et al., 2011). Human PARs consist of PAR1 and PAR2 and they have specific structural characteristics. In PAR1, all the genes that have been discovered to date escape the inactivation of the X chromosome and regulate growth in the body (Barrenäs et al., 2000). Previous research has reported that people with X chromosome escape genes on the short p-arm are more susceptible to ear and hearing issues (Barrenäs et al., 2000). SLC25A6 is typically an X-chromosome escape gene, which is an ADP/ATP translocase that is ubiquitously expressed at levels that are proportional to the respiratory activity of the tissue (Clémençon et al., 2013). As a core component of the mitochondrial permeability transition pore, SLC25A6 can greatly induce apoptosis of cultured human-derived cells (Silva-Marrero et al., 2017). This gene is expressed in immune, nervous, musculoskeletal, internal, secretory, and reproductive systems, especially overexpressed in the nasal epithelium, brain, and frontal cortex; most of these organs are TS-related. SLC25A6 is also expressed in the inner ear hair cell triggers apoptosis which is associated with sensorineural hearing loss (Jang and Lee, 2006). From Coexpedia database (http://www.coexpedia.org/), the top five syn-expressed genes of SLC25A6 are RPL8, RPS9, PHB2, RPL15, and RPS16. But whether those genes contributed to the TS pathomechanism or relevant developmental processes still need further research. Although there was no other research to find the members of the mitochondrial carrier subfamily involved in the pathogenesis of TS. However, there was a clinical study suggested differences in the metabolic demands of exercise between patients with TS and healthy control subjects (Wells et al., 2013). It proposes that mitochondrial metabolism may be involved in the TS pathophysiological processes. However, more research in this area is still needed. For TS patients, timely diagnosis and early treatment are important factors when it comes to improving the prognosis. It is important to explore potential biomarkers as well as prognostic indicators for accurate diagnosis and intervention. We suggested that the SLC25A6 gene as a potential candidate of the biomarker for otologic disorders in TS. It is meaningful that detect SLC25A6 in PBMCs for suspicious patients before genetic tests. In summary, the SLC25A6 gene is a strong candidate for explaining features regarding hearing loss in TS, but follow-up research is still needed.

Previous studies suggested that immune system disorders in females with TS included lower levels of IgG, a lower percentage of CD4^+^ T cells and a smaller CD4^+^ T cell to CD8^+^ T cell ratio than were observed among healthy controls (Gawlik et al., 2018). Through immune cell infiltration assays, we found the same conclusion: TS is linked with immune response alterations. In addition to CD4^+^ T and CD8^+^ T cells, immune cells such as macrophages, dendritic cells, and Tregs are related to TSE. Moreover, the SLC25A6 gene plays a regulatory role in the immune system.

However, there are several potential shortcomings of this study. First, as the dataset is open to the public, the information (age, health condition, and the medication usage of the individuals) was unattainable. This missing information appeared to be a possible limitation. Second, our findings need to be validated by further studies, including real-time PCR, western blot, and immunohistochemistry assays.

In summary, our study offers a comprehensive bioinformatics analysis based on two combined gene expression datasets (GSE46687 and GSE58435) used to explore miRNA differential expression linked with the progression of otologic disorders in TS. Functional enrichment analyses demonstrated targeting inflammatory responses, epigenetic regulation, transcriptional processes, and phospholipid metabolism may be potential strategies for treating otologic disorders in TS. Moreover, several hub genes were identified, and this information can help identify novel biomarkers and therapeutic drugs for treating otologic disorders in TS. Importantly, using ML analyses, we identified a novel biomarker (SLC25A6) of the pathogenesis of ear and hearing problems in TS. In addition, TSE are associated with alterations of the immune response. However, it is necessary to conduct more molecular experiments to verify the results of this study.

## Data Availability

Publicly available datasets were analyzed in this study. This data can be found here: https://www.ncbi.nlm.nih.gov/geo/query/acc.cgi?acc=GSE46687, using code GSE46687; https://www.ncbi.nlm.nih.gov/geo/query/acc.cgi?acc=GSE58435, using code GSE58435; https://www.ncbi.nlm.nih.gov/geo/query/acc.cgi?acc=GSE32527, using code GSE32527.

## Glossary

TS: turner syndrome
DEGs: differentially expressed genes
TSEs: ear-related genes of TS
GEO: gene expression omnibus
CTD: comparative toxicogenomic database
GO: gene ontology
KEGG: the kyoto encyclopedia of genes and genomes
DO: disease ontology
GSEA: gene set enrichment analysis
GSVA: gene set variation analysis
PPI: protein-protein interaction
TFs: transcription factors
DGIdb: drug-gene interaction database
HTS: high-throughput sequencing
NGS: next-generation sequencing
RNA-seq: RNA sequencing
PAR: pseudoautosomal region
ML: machine-learning
logFC: log fold change
BP: biological process
CC: cellular component
MF: molecular function
STRING: search tool for the retrieval of interacting genes
MCC: maximal clique centrality
WP: WikiPathway
MSigDB: molecular signatures database
LASSO: least absolute shrinkage and selection operator
RFB: random forests and Boruta
XGBoost: extreme gradient boosting
ROC: receiver operating characteristic
AUC: receiver operating characteristic curve
SVM: support vector machine
RBF: radial basis function
FDR: false detection rate
DAVID: database for annotation, visualization, and integrated discovery
SMS: sphingomyelin synthase
TRIM: tripartite motif
