# Supplementary figures and images for: Integrative Analyses of Genes Associated With Otologic Disorders in Turner Syndrome
Source: Front Genet. 2022 Feb 22;13:799783. doi: 10.3389/fgene.2022.799783 (PMC8902304; doi:10.3389/fgene.2022.799783)

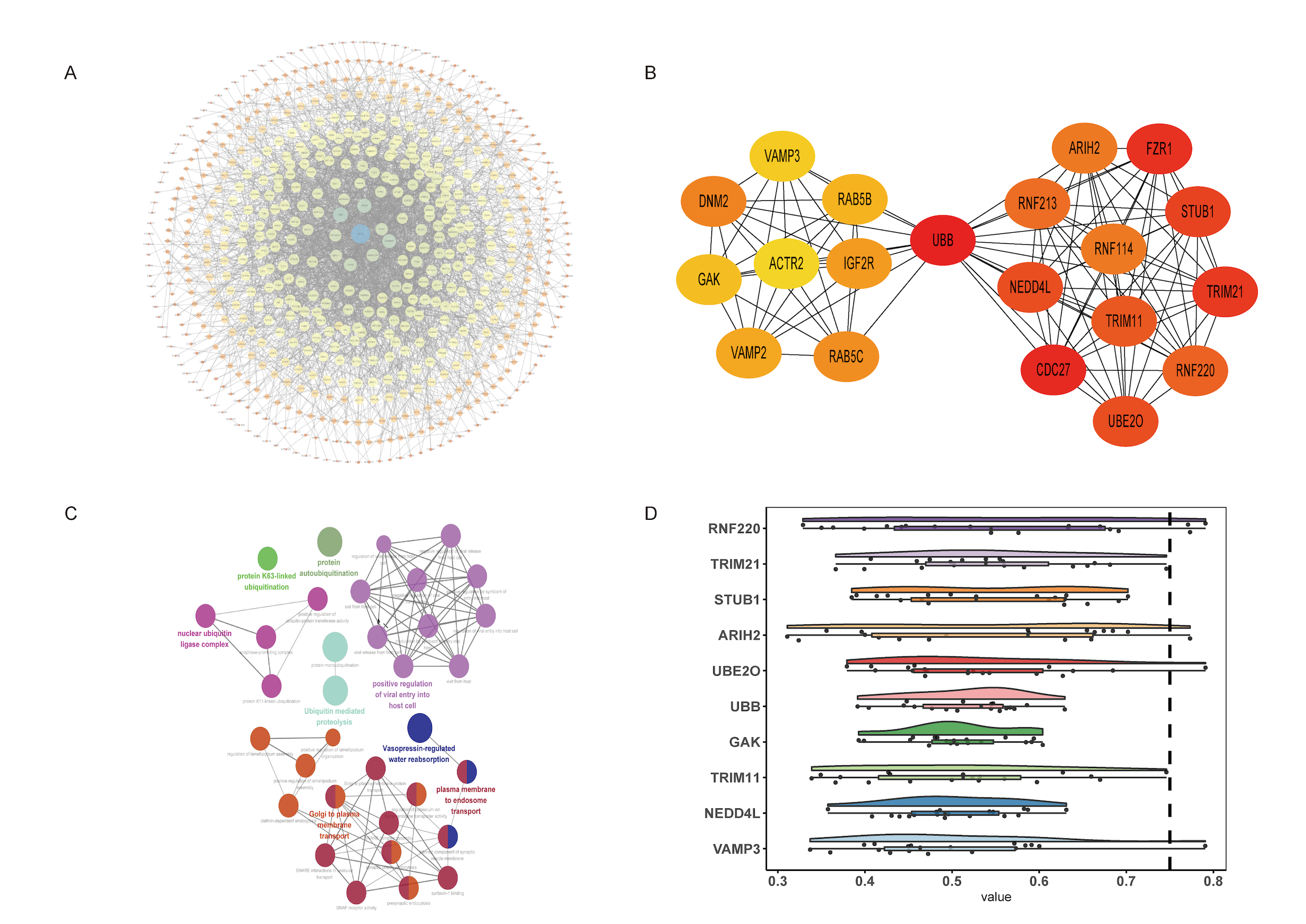

Supplement: Supplementary file 1 [file Image3.TIF]

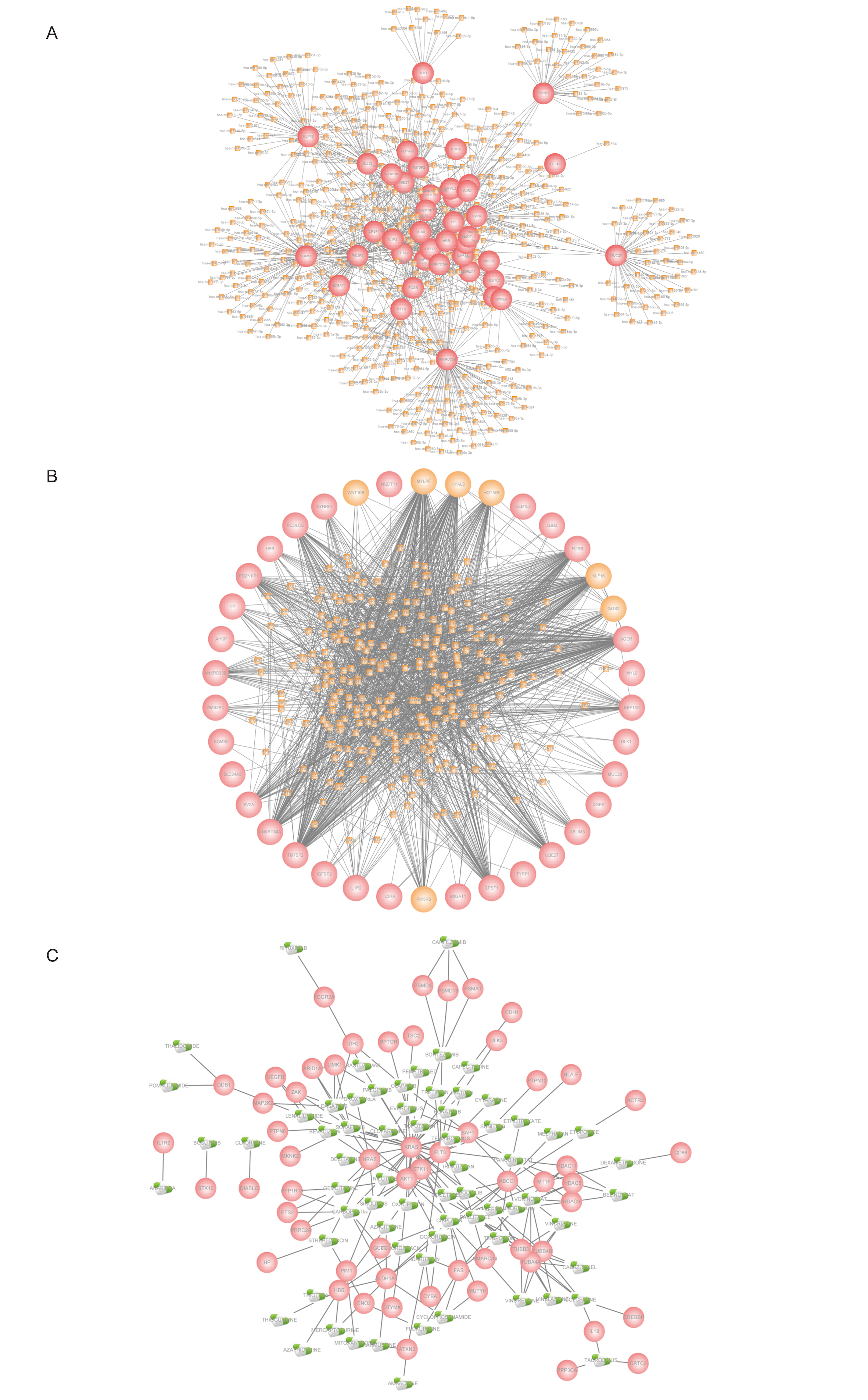

Supplement: Supplementary file 2 [file Image4.TIF]

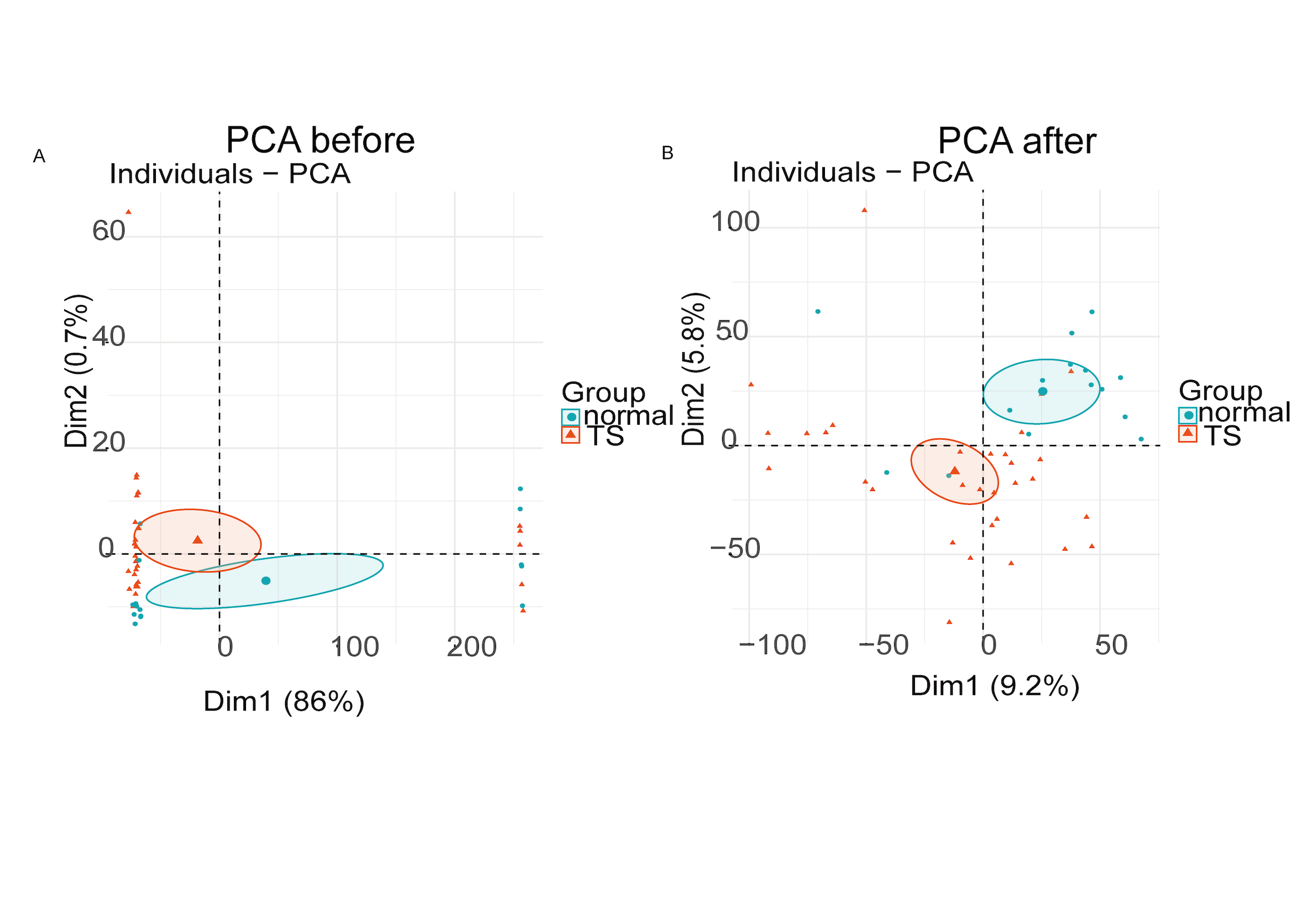

Supplement: Supplementary file 3 [file Image2.TIF]

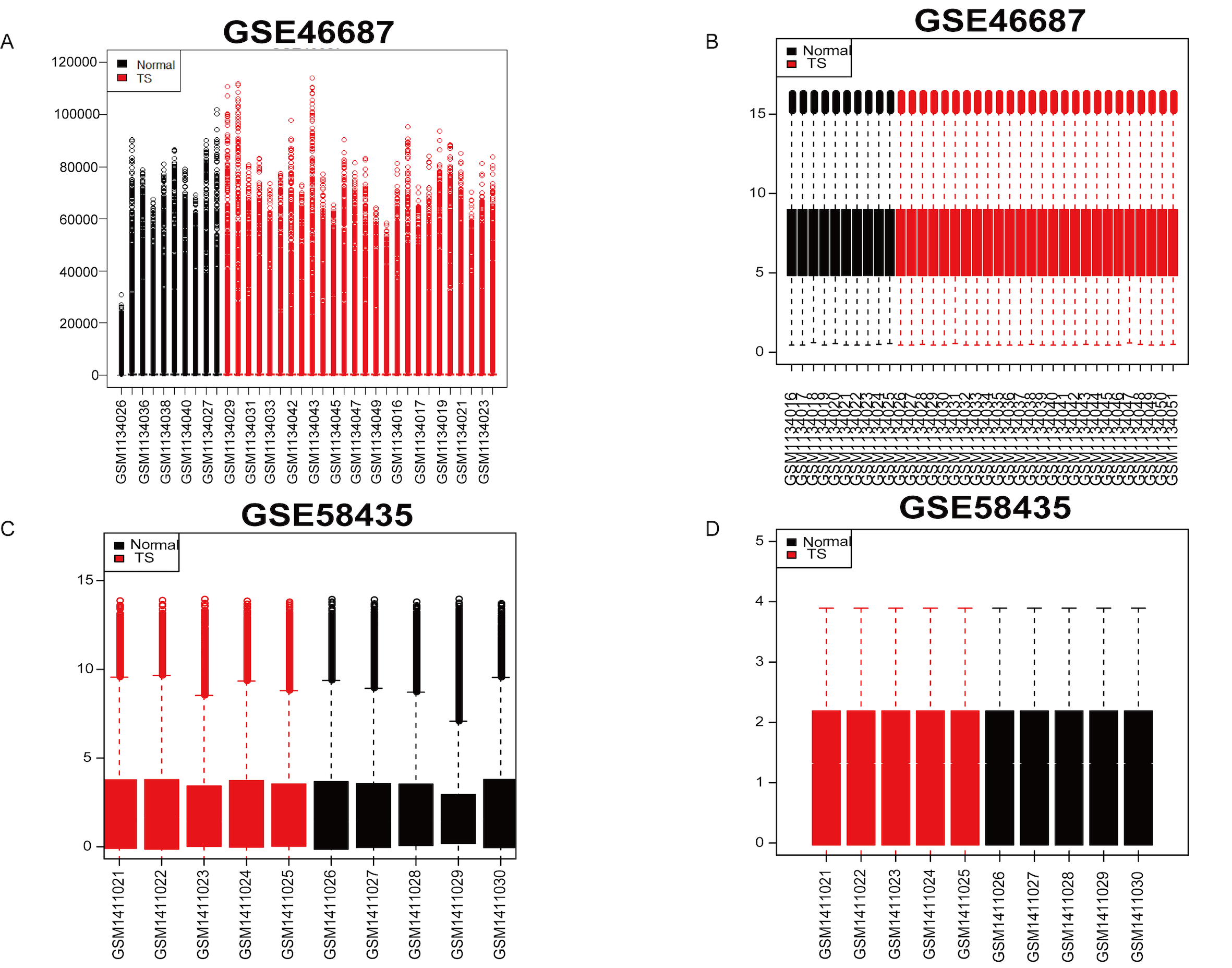

Supplement: Supplementary file 4 [file Image1.TIF]
