# Supplementary material for: Integrative Analyses of Genes Associated With Otologic Disorders in Turner Syndrome
Source: Front Genet. 2022 Feb 22;13:799783. doi: 10.3389/fgene.2022.799783 (PMC8902304; doi:10.3389/fgene.2022.799783)
Supplement: Supplementary file 5 [file DataSheet1.docx]

Supplementary Material

# Supplementary Tables

**Supplementary Table 1.** GO and KEGG enrichment analysis results.

| **ONTOLOGY** | **ID** | **Description** | **p.adjust** |
| --- | --- | --- | --- |
| BP | GO:0031647 | regulation of protein stability | 0.009000295 |
| BP | GO:0009896 | positive regulation of catabolic process | 0.010037771 |
| BP | GO:0042176 | regulation of protein catabolic process | 0.013833948 |
| BP | GO:0051090 | regulation of DNA-binding transcription factor activity | 0.014563149 |
| BP | GO:0016570 | histone modification | 0.014563149 |
| BP | GO:0016569 | covalent chromatin modification | 0.014563149 |
| BP | GO:0031331 | positive regulation of cellular catabolic process | 0.014563149 |
| BP | GO:0032507 | maintenance of protein location in cell | 0.022956158 |
| BP | GO:0072595 | maintenance of protein localization in organelle | 0.022956158 |
| BP | GO:0045732 | positive regulation of protein catabolic process | 0.024089777 |
| BP | GO:0045185 | maintenance of protein location | 0.026354261 |
| BP | GO:0019058 | viral life cycle | 0.026354261 |
| BP | GO:0032434 | regulation of proteasomal ubiquitin-dependent protein catabolic process | 0.026354261 |
| BP | GO:0006513 | protein monoubiquitination | 0.026354261 |
| BP | GO:0042129 | regulation of T cell proliferation | 0.027335934 |
| BP | GO:0042102 | positive regulation of T cell proliferation | 0.027335934 |
| BP | GO:0051091 | positive regulation of DNA-binding transcription factor activity | 0.027335934 |
| BP | GO:1902187 | negative regulation of viral release from host cell | 0.027335934 |
| BP | GO:0010498 | proteasomal protein catabolic process | 0.029067937 |
| BP | GO:0043161 | proteasome-mediated ubiquitin-dependent protein catabolic process | 0.030656913 |
| BP | GO:1903829 | positive regulation of cellular protein localization | 0.032935911 |
| BP | GO:0016049 | cell growth | 0.032935911 |
| BP | GO:1902369 | negative regulation of RNA catabolic process | 0.03443061 |
| BP | GO:0006338 | chromatin remodeling | 0.03611033 |
| BP | GO:0061136 | regulation of proteasomal protein catabolic process | 0.037023831 |
| BP | GO:0042098 | T cell proliferation | 0.038000288 |
| BP | GO:1901800 | positive regulation of proteasomal protein catabolic process | 0.040574041 |
| BP | GO:0001787 | natural killer cell proliferation | 0.040602333 |
| BP | GO:0001558 | regulation of cell growth | 0.041702051 |
| BP | GO:0043555 | regulation of translation in response to stress | 0.041702051 |
| BP | GO:0043489 | RNA stabilization | 0.041702051 |
| BP | GO:0033157 | regulation of intracellular protein transport | 0.043151034 |
| BP | GO:0050670 | regulation of lymphocyte proliferation | 0.043386971 |
| BP | GO:0006457 | protein folding | 0.043386971 |
| BP | GO:0032944 | regulation of mononuclear cell proliferation | 0.043386971 |
| BP | GO:1903364 | positive regulation of cellular protein catabolic process | 0.043386971 |
| BP | GO:1903362 | regulation of cellular protein catabolic process | 0.044895014 |
| BP | GO:1902373 | negative regulation of mRNA catabolic process | 0.044895014 |
| BP | GO:0051651 | maintenance of location in cell | 0.049053786 |
| CC | GO:0031301 | integral component of organelle membrane | 0.02548301 |
| CC | GO:0031300 | intrinsic component of organelle membrane | 0.02548301 |
| CC | GO:1905368 | peptidase complex | 0.02548301 |
| CC | GO:0005635 | nuclear envelope | 0.02548301 |
| CC | GO:0031248 | protein acetyltransferase complex | 0.02548301 |
| CC | GO:1902493 | acetyltransferase complex | 0.02548301 |
| CC | GO:0016514 | SWI/SNF complex | 0.02548301 |
| CC | GO:0030662 | coated vesicle membrane | 0.02548301 |
| CC | GO:0031965 | nuclear membrane | 0.02548301 |
| CC | GO:1904949 | ATPase complex | 0.030832927 |
| CC | GO:0000118 | histone deacetylase complex | 0.033254918 |
| CC | GO:0070603 | SWI/SNF superfamily-type complex | 0.044293576 |
| CC | GO:0030667 | secretory granule membrane | 0.046854139 |
| CC | GO:0005925 | focal adhesion | 0.046854139 |
| CC | GO:0005924 | cell-substrate adherens junction | 0.048575706 |
| CC | GO:0030055 | cell-substrate junction | 0.049839728 |
| CC | GO:0030660 | Golgi-associated vesicle membrane | 0.049839728 |
| MF | GO:0004407 | histone deacetylase activity | 0.017455614 |
| MF | GO:0033558 | protein deacetylase activity | 0.017455614 |
| MF | GO:0071889 | 14-3-3 protein binding | 0.017455614 |
| KEGG | hsa05132 | Salmonella infection | 0.005726783 |
| KEGG | hsa05135 | Yersinia infection | 0.005726783 |
| KEGG | hsa04144 | Endocytosis | 0.008273694 |
| KEGG | hsa05167 | Kaposi sarcoma-associated herpesvirus infection | 0.008273694 |
| KEGG | hsa04141 | Protein processing in endoplasmic reticulum | 0.011875033 |
| KEGG | hsa05170 | Human iTSunodeficiency virus 1 infection | 0.019010266 |
| KEGG | hsa05230 | Central carbon metabolism in cancer | 0.019010266 |
| KEGG | hsa04370 | VEGF signaling pathway | 0.019010266 |
| KEGG | hsa05231 | Choline metabolism in cancer | 0.019010266 |
| KEGG | hsa05203 | Viral carcinogenesis | 0.019010266 |
| KEGG | hsa05235 | PD-L1 expression and PD-1 checkpoint pathway in cancer | 0.024619759 |
| KEGG | hsa04660 | T cell receptor signaling pathway | 0.024938295 |
| KEGG | hsa04919 | Thyroid hormone signaling pathway | 0.027524132 |
| KEGG | hsa04145 | Phagosome | 0.027524132 |
| KEGG | hsa05221 | Acute myeloid leukemia | 0.028625376 |
| KEGG | hsa04662 | B cell receptor signaling pathway | 0.031642504 |
| KEGG | hsa05010 | Alzheimer disease | 0.039190826 |
| KEGG | hsa05131 | Shigellosis | 0.040786952 |
| KEGG | hsa05130 | Pathogenic Escherichia coli infection | 0.0409648 |
| KEGG | hsa04072 | Phospholipase D signaling pathway | 0.0409648 |
| KEGG | hsa04071 | Sphingolipid signaling pathway | 0.043506868 |
| KEGG | hsa05022 | Pathways of neurodegeneration - multiple diseases | 0.043506868 |
| KEGG | hsa05216 | Thyroid cancer | 0.043506868 |
| KEGG | hsa04210 | Apoptosis | 0.043506868 |
| KEGG | hsa04140 | Autophagy - animal | 0.043574218 |
| KEGG | hsa05100 | Bacterial invasion of epithelial cells | 0.043574218 |
| DO | umls:C0027708 | Nephroblastoma | 5.10E-05 |
| DO | umls:C0036920 | Sezary Syndrome | 5.72E-05 |
| DO | umls:C0020757 | Ichthyoses | 0.000141989 |
| DO | umls:C0020758 | Congenital ichthyosis | 0.000141989 |
| DO | umls:C0242656 | Disease Progression | 0.00014397 |
| DO | umls:C0476073 | Papillary neoplasm | 0.000349575 |
| DO | umls:C1334928 | Necrotic changes (finding) | 0.000360824 |
| DO | umls:C0685938 | Malignant neoplasm of gastrointestinal tract | 0.000423026 |
| DO | umls:C0026848 | Myopathy | 0.000575124 |
| DO | umls:C0037023 | Sialadenitis | 0.000578247 |

**Supplementary Table 2.** GSEA enrichment analysis result.

| **ONTOLOGY** | **ID** | **Description** | **p.adjust** |
| --- | --- | --- | --- |
| BP | GO:0046486 | glycerolipid metabolic process | 0.001519757 |
| BP | GO:0042060 | wound healing | 0.00295858 |
| BP | GO:0009611 | response to wounding | 0.002985075 |
| BP | GO:0044706 | multi-multicellular organism process | 0.00317965 |
| BP | GO:0030855 | epithelial cell differentiation | 0.00456621 |
| BP | GO:0048545 | response to steroid hormone | 0.004615385 |
| BP | GO:0045333 | cellular respiration | 0.00536193 |
| BP | GO:0040008 | regulation of growth | 0.005578801 |
| BP | GO:0050878 | regulation of body fluid levels | 0.00617284 |
| BP | GO:0044093 | positive regulation of molecular function | 0.00656168 |
| BP | GO:0048518 | positive regulation of biological process | 0.006833713 |
| BP | GO:0043085 | positive regulation of catalytic activity | 0.007853403 |
| BP | GO:0048522 | positive regulation of cellular process | 0.007874016 |
| BP | GO:0007596 | blood coagulation | 0.008 |
| BP | GO:0007599 | hemostasis | 0.008 |
| BP | GO:0050817 | coagulation | 0.008 |
| BP | GO:0051254 | positive regulation of RNA metabolic process | 0.008053691 |
| BP | GO:0032870 | cellular response to hormone stimulus | 0.008486563 |
| BP | GO:0044703 | multi-organism reproductive process | 0.008595989 |
| BP | GO:1902680 | positive regulation of RNA biosynthetic process | 0.009259259 |
| BP | GO:1903508 | positive regulation of nucleic acid-templated transcription | 0.009259259 |
| BP | GO:0050789 | regulation of biological process | 0.010044643 |
| BP | GO:0015980 | energy derivation by oxidation of organic compounds | 0.011080332 |
| BP | GO:0009725 | response to hormone | 0.011157601 |
| BP | GO:0010557 | positive regulation of macromolecule biosynthetic process | 0.01164295 |
| BP | GO:0009719 | response to endogenous stimulus | 0.011968085 |
| BP | GO:0045944 | positive regulation of transcription by RNA polymerase II | 0.012 |
| BP | GO:0051128 | regulation of cellular component organization | 0.012658228 |
| BP | GO:0030168 | platelet activation | 0.012924071 |
| BP | GO:0033674 | positive regulation of kinase activity | 0.015759312 |
| BP | GO:0045935 | positive regulation of nucleobase-containing compound metabolic process | 0.015831135 |
| BP | GO:0051224 | negative regulation of protein transport | 0.015831135 |
| BP | GO:1904950 | negative regulation of establishment of protein localization | 0.015831135 |
| BP | GO:0051051 | negative regulation of transport | 0.015873016 |
| BP | GO:0001558 | regulation of cell growth | 0.016034985 |
| BP | GO:0000003 | reproduction | 0.016460905 |
| BP | GO:0022414 | reproductive process | 0.016460905 |
| BP | GO:1901654 | response to ketone | 0.017770598 |
| BP | GO:0009891 | positive regulation of biosynthetic process | 0.018252934 |
| BP | GO:0031328 | positive regulation of cellular biosynthetic process | 0.018324607 |
| BP | GO:0051048 | negative regulation of secretion | 0.018469657 |
| BP | GO:1903531 | negative regulation of secretion by cell | 0.018469657 |
| BP | GO:0001101 | response to acid chemical | 0.0192 |
| BP | GO:0045893 | positive regulation of transcription, DNA-templated | 0.019633508 |
| BP | GO:0002791 | regulation of peptide secretion | 0.021084337 |
| BP | GO:0050708 | regulation of protein secretion | 0.021084337 |
| BP | GO:0003006 | developmental process involved in reproduction | 0.02130898 |
| BP | GO:0006355 | regulation of transcription, DNA-templated | 0.022058824 |
| BP | GO:0050794 | regulation of cellular process | 0.022371365 |
| BP | GO:0031325 | positive regulation of cellular metabolic process | 0.022592152 |
| BP | GO:0007565 | female pregnancy | 0.022617124 |
| BP | GO:0071495 | cellular response to endogenous stimulus | 0.022911051 |
| BP | GO:0006091 | generation of precursor metabolites and energy | 0.025236593 |
| BP | GO:1903506 | regulation of nucleic acid-templated transcription | 0.025516403 |
| BP | GO:2001141 | regulation of RNA biosynthetic process | 0.025516403 |
| BP | GO:0007167 | enzyme linked receptor protein signaling pathway | 0.026990553 |
| BP | GO:0016570 | histone modification | 0.027298851 |
| BP | GO:1901615 | organic hydroxy compound metabolic process | 0.027700831 |
| BP | GO:0051173 | positive regulation of nitrogen compound metabolic process | 0.031287605 |
| BP | GO:0032504 | multicellular organism reproduction | 0.032835821 |
| BP | GO:0048609 | multicellular organismal reproductive process | 0.032835821 |
| BP | GO:0006351 | transcription, DNA-templated | 0.032846715 |
| BP | GO:0009893 | positive regulation of metabolic process | 0.033412888 |
| BP | GO:0017144 | drug metabolic process | 0.035031847 |
| BP | GO:0010817 | regulation of hormone levels | 0.03601108 |
| BP | GO:0090092 | regulation of transmembrane receptor protein serine/threonine kinase signaling pathway | 0.037441498 |
| BP | GO:0051252 | regulation of RNA metabolic process | 0.03823178 |
| BP | GO:0007264 | small GTPase mediated signal transduction | 0.038404727 |
| BP | GO:0006357 | regulation of transcription by RNA polymerase II | 0.038910506 |
| BP | GO:0009306 | protein secretion | 0.039473684 |
| BP | GO:0006650 | glycerophospholipid metabolic process | 0.040561622 |
| BP | GO:0032774 | RNA biosynthetic process | 0.040963855 |
| BP | GO:0097659 | nucleic acid-templated transcription | 0.040963855 |
| BP | GO:0008284 | positive regulation of cell proliferation | 0.042075736 |
| BP | GO:0060429 | epithelium development | 0.04264099 |
| BP | GO:1903530 | regulation of secretion by cell | 0.042763158 |
| BP | GO:0061458 | reproductive system development | 0.043076923 |
| BP | GO:0065007 | biological regulation | 0.043381535 |
| BP | GO:0048608 | reproductive structure development | 0.04524181 |
| BP | GO:0002790 | peptide secretion | 0.045901639 |
| BP | GO:0007265 | Ras protein signal transduction | 0.046296296 |
| BP | GO:2000112 | regulation of cellular macromolecule biosynthetic process | 0.047789725 |
| BP | GO:0045597 | positive regulation of cell differentiation | 0.047819972 |
| BP | GO:0006366 | transcription by RNA polymerase II | 0.048717949 |
| CC | GO:0044297 | cell body | 0.001455604 |
| CC | GO:0005743 | mitochondrial inner membrane | 0.002840909 |
| CC | GO:0036477 | somatodendritic compartment | 0.004243281 |
| CC | GO:0043025 | neuronal cell body | 0.00443787 |
| CC | GO:0042995 | cell projection | 0.005249344 |
| CC | GO:0030667 | secretory granule membrane | 0.006079027 |
| CC | GO:0031224 | intrinsic component of membrane | 0.006097561 |
| CC | GO:0016021 | integral component of membrane | 0.006134969 |
| CC | GO:0120025 | plasma membrane bounded cell projection | 0.012016021 |
| CC | GO:0031966 | mitochondrial membrane | 0.013029316 |
| CC | GO:0097458 | neuron part | 0.013297872 |
| CC | GO:0044455 | mitochondrial membrane part | 0.015831135 |
| CC | GO:0005740 | mitochondrial envelope | 0.015923567 |
| CC | GO:0044463 | cell projection part | 0.016348774 |
| CC | GO:0120038 | plasma membrane bounded cell projection part | 0.016348774 |
| CC | GO:0005576 | extracellular region | 0.017899761 |
| CC | GO:0098798 | mitochondrial protein complex | 0.018716578 |
| CC | GO:0044425 | membrane part | 0.023255814 |
| CC | GO:0044429 | mitochondrial part | 0.033457249 |
| CC | GO:0019866 | organelle inner membrane | 0.049275362 |
| MF | GO:0005488 | binding | 0.004581901 |
| MF | GO:0060089 | molecular transducer activity | 0.006557377 |
| MF | GO:0000977 | RNA polymerase II regulatory region sequence-specific DNA binding | 0.010028653 |
| MF | GO:0001012 | RNA polymerase II regulatory region DNA binding | 0.010028653 |
| MF | GO:0038023 | signaling receptor activity | 0.013157895 |
| MF | GO:0043565 | sequence-specific DNA binding | 0.016348774 |
| MF | GO:0004888 | transmembrane signaling receptor activity | 0.019047619 |
| MF | GO:0000976 | transcription regulatory region sequence-specific DNA binding | 0.025034771 |
| MF | GO:0003690 | double-stranded DNA binding | 0.025210084 |
| MF | GO:1990837 | sequence-specific double-stranded DNA binding | 0.027510316 |
| MF | GO:0003677 | DNA binding | 0.032808399 |
| MF | GO:0001067 | regulatory region nucleic acid binding | 0.034867503 |
| MF | GO:0044212 | transcription regulatory region DNA binding | 0.034867503 |
| MF | GO:0001228 | DNA-binding transcription activator activity, RNA polymerase II-specific | 0.0448 |
| MF | GO:0005515 | protein binding | 0.045758929 |
| pathway | WP_ANGIOPOIETIN_LIKE_PROTEIN_8_REGULATORY_PATHWAY | WP_ANGIOPOIETIN_LIKE_PROTEIN_8_REGULATORY_PATHWAY | 0.00755287 |
| pathway | KEGG_CYTOKINE_CYTOKINE_RECEPTOR_INTERACTION | KEGG_CYTOKINE_CYTOKINE_RECEPTOR_INTERACTION | 0.00887574 |
| pathway | REACTOME_SEPARATION_OF_SISTER_CHROMATIDS | REACTOME_SEPARATION_OF_SISTER_CHROMATIDS | 0.022922636 |
| pathway | REACTOME_PHOSPHOLIPID_METABOLISM | REACTOME_PHOSPHOLIPID_METABOLISM | 0.028700906 |
| pathway | PID_P53_DOWNSTREAM_PATHWAY | PID_P53_DOWNSTREAM_PATHWAY | 0.032258065 |
| pathway | REACTOME_G_ALPHA_Q_SIGNALLING_EVENTS | REACTOME_G_ALPHA_Q_SIGNALLING_EVENTS | 0.034743202 |
| pathway | WP_INSULIN_SIGNALING | WP_INSULIN_SIGNALING | 0.039156627 |
| pathway | WP_B_CELL_RECEPTOR_SIGNALING_PATHWAY | WP_B_CELL_RECEPTOR_SIGNALING_PATHWAY | 0.044410413 |

# 2． Supplementary Figure Legends

**Supplementary Figure 1.** Sample preprocessing. (**A**), (**B**): Gene expression data from the GSE46687 dataset before and after normalization. (**C**), (**D**): Gene expression data from the GSE58435 dataset before and after normalization. The abscissa represents the sample, and the ordinate indicates the expression values of the genes. The red line in the box plot denotes the TS tissue. The black line in the box plot denotes the normal tissue. TS, Turner syndrome.

**Supplementary Figure 2.** Principal component analysis (PCA) before and after batch correction of the merged data sets. (**A**): PCA before batch correction, (**B**): PCA after batch correction. The red triangles in the PCA plot represent the TS tissue. The blue circles in the PCA plot represent the normal tissue.

**Supplementary Figure 3.** PPI network and identification and visualization of hub genes. (**A**): The ear-related TS gene PPI network was analyzed with STRING software, with each node representing a gene and each edge representing an interaction. (**B**): The hub genes in the PPI network were identified utilizing the MCC algorithm. The hub genes with the highest 20 scores. (**C**): Gene functional enrichment analysis of the hub module was performed using ClueGO and CluePedia within the Cytoscape framework. (**D**): Identification of the hub genes.

**Supplementary Figure 4.** Target gene‐miRNA network of TSEs, TF‐target gene network of TSEs, and drug-interaction network of DEGs. (**A**): The target gene‐miRNA network. Genes are shown as red circle nodes, and miRNAs are shown as yellow diamond nodes. (**B**): The TF‐target gene network. Genes are shown as red circle nodes, genes and TFs are shown as yellow circle nodes, and TFs are shown as yellow diamond nodes. (**C**): The drug-interaction network. Genes are shown as red circle nodes, and drugs are shown as green drug nodes.
